# Evaluation of the Use of Herbs, Spices, and Supplements in the Hispanic/Latino/Latina Population in Kokomo, Indiana, U.S.A.

**DOI:** 10.1007/s10900-025-01546-7

**Published:** 2025-12-21

**Authors:** Kim Mossburg, Cheryl Moore-Beyioku, J. R. Pico, Christopher Caruvana, Lalatendu Acharya

**Affiliations:** Indiana University Kokomo, 2300 S. Washington Street, Kokomo, IN 46902 USA

**Keywords:** Supplements, Disease, Herbs, Spices, Population

## Abstract

Although the Hispanic/Latino/Latina immigrant population group has been documented to use a higher number of herbs, spices and supplements, research findings on this topic are still insufficient. This study aimed to evaluate the supplements chosen by Hispanic/Latino/Latina immigrants in relation to their evidence for use in the literature as well as the number of herbs and spices used. This cross-sectional study utilized convenience sampling to survey 62 Hispanic/Latino/Latina immigrants in central Indiana. The research collected data regarding herbs and spice consumption, supplement usage and beliefs, therapeutic diet practices, disease history, and food frequency information. Dietary supplement use was more prevalent among females and individuals with a high school education. A statistically significant difference between male and female participants was observed regarding the use of green tea supplements, as well as all herbal products and single vitamins combined (*p <.001*). Significant associations were also found when comparing individuals with bone and joint (*p =.030*), cardiovascular (*p =.024*), gastrointestinal (*p =.031*), and endocrine diseases (*p =.020*) to those adhering to a special diet. There was a lack of evidence supporting supplement use based on research findings. These results suggest for the need of supplement and therapeutic diet use education in the Hispanic/Latino/Latina population residing in the United States. Further research is needed to advance these findings.

## Introduction

The consumption of micronutrients in the United States is associated with shortfalls in several areas. Analysis of National Health and Nutrition Examination Survey (NHANES) data from 2003 to 2018 indicates adult populations commonly fail to meet recommended levels of choline, calcium, magnesium, potassium, and vitamins A, C, D, E, and K [1]. These deficiencies, alongside considerations for disease prevention and management are primary reasons for the use of dietary supplements [2].

User motivations for supplement use have traditionally been linked to health enhancement, with healthcare providers participating in fewer than 25% of supplement recommendations [3]. Current surveys indicate that dietary supplement guidance from healthcare professionals differs across professional groups [4]. During the COVID-19 pandemic, vitamins C and zinc emerged as the most commonly recommended supplements by healthcare professionals, which may have influenced supplement consumption within this population [5].

Concerns persist regarding the use of supplements without sufficient understanding, ingredient safety, and the reliability of third-party testing organizations. Although dietary supplements are regulated and prohibited from making disease treatment claims [6], unauthorized assertions may still influence consumer purchasing decisions [7]. In addition, undeclared contaminants, including certain drugs found in supplements, may contribute additional effects for consumers [8].

The recommendation of specific dietary supplements for certain disease states supports perhaps a few supplements depending on the condition. Supplements will be reviewed based on this study population’s intake. The current recommendations for cardiovascular disease have varying levels of evidence which depend on the specific problem. An et al., in a large systematic review found that the use of curcumin, catechin, vitamin D, and omega-3 had moderate to high quality evidence for the reduction of risk factors associated with cardiovascular disease. The use of vitamins C, D and E, however, was not effective on cardiovascular disease or diabetes type 2 risks [9]. Despite these findings, recent studies question the use of fish oil supplements due to a possible risk of atrial fibrillation and stroke [10]. Previous observational studies looking at cardiovascular disease and vitamin D support lower vitamin D levels contributing to higher cardiovascular disease risks [11–13], many of the more recent clinical trials provide possible support for these findings [14–17] although further evidence is still required. The use of catechin is supported by several systematic reviews in relation to metabolic syndrome [18–20]. Curcumin has the same emerging support in systematic reviews [21–24]. Garlic use has been suggested to play a supportive role in hypertension [25, 26] as well as the possible reduction of hyperlipidemia [27, 28].

Support for dietary supplements in bone and joint disease reflects newer evidence in support of vitamins D and K together for osteoporosis [29, 30]. Adequate calcium with lower supplementary doses is now recommended secondary to the evidence showing that only around 500 mg can be absorbed at one time [31] and the possible increased risks of stroke and myocardial infarction from high dose calcium. Explanations seem to support an imbalance between vitamins K and D for the deposition of calcium into the vascular system [32, 33]. The use of curcumin has emerging evidence supporting its use in arthritic conditions [34, 35]. Ginger, as well as curcumin has been shown to be supportive in the treatment of osteoarthritis [36, 37] in some studies but not in others [38, 39] which may be due to the variability of ginger compounds [40].

Gastrointestinal support from dietary supplements as well as for endocrine associated diseases will vary widely depending upon the origin of the problem. Although emerging, curcumin/turmeric has been suggested to have a beneficial impact on gastrointestinal disease, specifically irritable bowel syndrome in several reviews [41, 42]. Other gastrointestinal conditions were unclear [43]. Probiotics were not listed in the supplements from participants and are therefore not included here.

Hypothyroidism was a prominent listing for the endocrine participants as far as diagnosis; however, supplements reported for this and other endocrine conditions by participants were not evidence-based. The endocrine group will not be addressed in the supplement part of the analysis.

Given the ongoing concerns regarding the evidence-based use of dietary supplements within the general population, and the notable lack of data pertaining to the Hispanic/Latino/Latina population, this article presents an observational analysis of dietary supplement intake among Hispanic/Latino/Latina people in central Indiana. Furthermore, it examines the role of herbs and spices within this group, specifically in relation to supplement consumption and therapeutic diet use.

## Materials and methods

### Survey

The data from the herb and spice intake form, as well as disease history information provided at the conclusion of the food frequency questionnaire, were analyzed for this report. Data items were collected between April 2021 and February 2022. Demographic details such as age, ethnicity, gender, education level, and duration of residence in the United States were collected. Survey instruments were available in both English and Spanish; bilingual professionals translated the English questionnaires into Spanish and subsequently back-translated them to English to eliminate inconsistencies. The selection of herbs and spices reflected those most commonly used within Hispanic/Latino/Latina communities, totaling 20 distinct items. Participants reported their usage frequency for each herb or spice as daily, weekly, or monthly. Diseases were categorized by body systems, and a therapeutic diet was defined as one that is scientifically recommended for the management of a specific health condition.

### Participants

The IRB-approved study received exempt status, and participants were recruited from surrounding communities near Indiana University Kokomo in collaboration with the university’s Hispanic Center. Eligibility criteria included Hispanic/Latino/Latina adults aged 18 years or older who were able to complete a paper survey and could consume standard food and beverages.

### Statistical Analysis

Survey data were entered into SPSS (version 29, 2022; IBM Inc.). All analyses utilized a significance threshold of *p* =.05. Figures were generated using Microsoft Excel (version 2019). Descriptive statistics were presented as counts and percentages where appropriate. Diseases with the highest representation were selected for analysis, and comparisons were conducted based on demographic criteria.

Given the non-parametric nature of the nominal data, as indicated by violation of the normality assumption in the Shapiro-Wilk test (*p* <.05), the Pearson Chi-square test was used to assess differences in disease states with respect to demographics, supplements, and special diets, as well as between special diets and specific diseases. The relationship between supplements and demographics, as well as the use of herbs, spices, and supplements, was analyzed using the Kruskal-Wallis test. This test was chosen due to its suitability for evaluating differences among three or more sub-groups when the dependent variable is not normally distributed, as determined by the Shapiro-Wilk test.

## Results

The study population ranged in age between 18 and 79. Table 1. summarizes the baseline characteristics in relation to gender, age, education, and ethnicity and representation of the most prevalent disease states. The country of origin in the bone and joint category (*p =.001*) was the only group not equally represented. The rest of the groups were similarly distributed.

**Table 1.** Demographics of those with select disease states

n=62	CVD	P	** Bone/Jt.**	P	GI	P	**Endocrine**	P
	N/%		N/%		N/%		N/%	
**Gender**		.356		.378		.169		*.451*
Male	3 (4.84)		2 (3.23)		2 (3.23)		4 (6.45)	
Female	2 (3.32)		5 (8.06)		8 (12.90)		6 (9.68)	
**Age**		.145		.026		.916		.078
18-40	1 (1.61)		1 (1.61)		5 (8.06)		2 (3.23)	
40+	4 (6.45)		6 (9.68)		4 (6.45)		6 (9.68)	
**Education**		.523		.183		.54		.44
<high school	1 (1.61)		0		1 (1.61)		2 (3.23)	
High School	2 (3.23)		5 (8.06)		5 (8.06)		5 (8.06)	
University	2 (3.23)		1 (1.61)		3 (4.84)		2 (3.23)	
**Country of origin**		.062		.001		.503		.301
Argentina	1 (1.61)		2 (3.23)		1 (1.61)		1 (1.61)	
Colombia	2 (3.23)		1 (1.61)		4 (6.45)		5 (8.06)	
Mexico	1 (1.61)		0		1 (1.61)		1 (1.61)	
Mixed	0		0		0		0	
Panama	0		0		0		0	
Peru	0		3 (4.84)		2 (3.23)		0	
Spain	1 (1.61)		1 (1.61)		0		0	
Venezuela	0		0		1 (1.61)		2 (3.23)	

Similar distributions were observed in the range of supplements taken in the gender, age, and ethnicity groups (Table 2).

**Table 2:** Number of supplements taken in the participants with diseases

n=62	Total Sample	Total Taking/Not Taking Supplements	0	1	2	3	4	5	6	7+
			N	N	N	N	N	N	N	N
**Gender** (*p* <.001) Male Female	23 36	15/8 30/5	8 5	6 8	2 4	4 6	2 4	1 2	0 3	0 3
**Age Group** (*p*=.207) 18–40 years 40+ years	36 23	26/10 19/3	10 3	9 4	3 3	9 2	3 3	1 2	0 3	1 2
**Ethnicity** (*p*=.019) Argentina Colombia Mexico Mixed Panama Peru Spain Venezuela	3 21 14 4 2 7 1 6	1/2 18/3 11/3 3/1 1/1 6/0 1/0 4/2	2 3 3 1 1 0 0 2	0 7 3 0 1 1 0 1	0 2 3 0 0 1 0 0	0 6 2 0 0 1 0 2	0 1 0 1 0 3 1 0	1 1 0 1 0 0 0 0	0 1 1 0 0 0 0 1	0 0 2 1 0 0 0 0
**Education** (*p* <.001) < High school High school University	8 28 19		3 7 1	1 8 5	2 2 2	1 5 5	0 3 3	0 2 1	0 1 1	0 0 2

Pearson Chi-square

Distributions of supplement use by gender and education level were predominantly non-significant, except for turmeric/curcumin intake among individuals holding a high school diploma. These findings indicate that, overall, gender and education did not substantially affect patterns of supplement usage (Table 2).

Analysis of the distribution of selected herbs and individual vitamins by gender and education revealed significant differences, except for vitamin C, which showed no association with education levels. Vitamin C was identified as the most commonly used single vitamin among participants. Seven individuals reported using vitamin E. Multivitamin use was reported by 23 participants (37.1% of the total), comprising 34.78% males and 60.87% females. Within this cohort, 15 participants consumed separate herbal supplements, and some used more than one, with garlic being the most prevalent (*n* = 10), followed by turmeric (*n* = 6).

**Table 3:** Most frequent supplement prevalence according to subject demographics

n=62	Green tea	Turmeric/Curcumin	Garlic	Vitamin C	Vitamin D
	N	N	N	N	N
**Gender** ***p-value*** Male Female	*p* <.001 1 8	*p* <.001 (across all herbs) 1 7	2 7	*p* <.001 (across all single vitamins) 10 14	2 9
**Education** ***p-value*** < High School Education	*p*=.011 0 2 5	*p*=.011 (across all herbs) 1 2 2	1 2 4	*p*=.177 (across all single vitamins) 2 11 9	0 1 8

Chi-Square was used for the p-value

Missing data account for number inconsistencies

The routine consumption of herbs and spices has been linked with health benefits, with existing evidence indicating that increased intake may confer enhanced health protection [44]. In Table 4, the total number of supplements was compared to the number of herbs and spices used daily. Participants who consumed six different herbs or spices per day exhibited the greatest overall use of supplements. This number contains participants in multiple disease groups. In this study, no significant differences were observed between daily herb and spice use and supplement use among the disease groups. However, a significant association was found between the number of different herbs used weekly and individuals who did or did not use supplements (*p* =.028). Most participants (20 out of 24) perceived herbs as beneficial to health, which was significantly associated with the use of turmeric in foods Χ^2^ (6, *n* = 33) = 16.859, *p* =.010. Although this finding was unexpected, it is reasonable considering the popular use of turmeric due to its perceived health benefits, however only eight participants took the supplemental form of turmeric/curcumin.

**Table 4:** Weekly herbs and spices used in relation to total number of supplements in disease group

n=29	Herbs and spices used weekly used	Total number of supplements used	*P*-value
	Number	Number	
**Disease Group** Cardiovascular Bone and joint Endocrine Gastrointestinal	9 6 10 8	9 5 9 6	*0.028* Number of herbs used weekly in those taking supplements or none

Kruskal-Wallis

Special diets are commonly recommended for various disease states. Analysis indicated no statistically significant differences among bone and joint, cardiovascular, gastrointestinal, and endocrine groups regarding adherence to special diets (Table 5). Six participants from the disease cohort reported following a special diet, comprising four females and two males. The highest incidence of dietary adherence was observed in individuals with cardiovascular disease (*n* = 3), followed by those with bone and joint conditions (*n* = 2), and gastrointestinal disorders (*n* = 1). Across the entire study population, significant correlations were found between following a special diet and both education level (*p* =.005) and gender group (*p* <.001). While no significant differences were observed across age groups, participants with higher levels of education were more likely to follow special diets.

**Table 5:** Participants using a special diet with specific diseases in disease group and according to education level and gender in whole study group

Disease Group	Follows special diet	Did not follow special diet	*P*-value
	*N*/%	*N*/%	
*Percents reflect individual groups*			
Bone and joint	2 (25)	6 (75)	.*030*
Cardiovascular	2 (25)	6 (75)	.*024*
Gastrointestinal disease	5 (62.5)	3 (37.5)	*0.031*
Endocrine disease	0	8 (100)	.*020*
*Percents reflect whole study group (n=62)* **Education Level** *p*=.005 < High school education High School education University education	0 3 (4.84) 7 (11.29)	8 (100) 24 (38.71) 12 (19.35)	
**Gender** *p*<.001 Male Female	2 (3.23) 8 (12.90)	21 (33.87) 27 (43.55)	
**Age Group** *p*=.551 18–40 years 40+	5 (8.06) 5 (8.06)	30 (48.39) 18 (29.03)	

Pearson Chi-Square

*Missing data is responsible for the number of inconsistencies

Participant motivations for supplement use among individuals with diseases exhibited variation across disease states, though these differences were not highly significant. The primary reason cited was nutrient deficiency; several participants expressed concerns that their dietary intake did not adequately provide necessary nutrients to maintain good health. Additional reasons included pain management, osteoporosis prevention, general health support, and increased energy.

Assessment of supplement selection in relation to specific disease categories indicated gaps between participant choices and evidence-based recommendations. The endocrine category was excluded, as no participants reported taking supplements demonstrated to benefit the majority of endocrine conditions. Accompanying each section is the percentage of participants who selected supplements with some evidence of therapeutic benefit for their respective disease state. Specifically, two participants chose supplements linked to potential cardiovascular benefits (33.3% of this group). For bone and joint disorders, two different participants each used a supplement that may be advantageous for these conditions (16.7% per individual in the group). Within the gastrointestinal category, one participant utilized a supplement with emerging evidence for efficacy (12.5% of this group).

## Discussion

Dietary supplement use is widespread across various populations, yet evidence-based use remains a significant concern. This study found that among participants with disease states, only a small proportion were consuming supplements with demonstrated benefits for their respective conditions (ranging from 12.5% to 33.3% per disease category). The analysis did not account for potential adverse effects of other supplements used. Previous research has indicated increased supplement consumption among highly educated, older Caucasian females with normal body weight and elevated fruit and vegetable intake [45, 46]. Data on Hispanic populations similarly report high rates of supplement use [47–49], which is consistent with our findings among those with disease. For instance, one participant reported the use of three single vitamins and two herbs. The highest level of supplement intake was documented in an older participant, comprising four single vitamins, two minerals, two herbs, and one antioxidant.

A separate study evaluating herbal use within the Women Infant Children (WIC) program highlighted the necessity of herbal education among Hispanics [50], as use of herbals has been reported to surpass that of medications in Hispanic households [51]. Our findings parallel this observation, with only 19 medications reported among all study participants (*n* = 62). Participants held positive beliefs regarding the health benefits of herbal products; however, these attitudes did not correspond with the quantity of supplements consumed. Garlic (24.2%) and green tea (18.2%) emerged as the most frequently used medicinal herbal supplements within the disease group of this population. These results diverge from those of Malika et al., who reported cinnamon, chamomile, aloe vera, key lime, and spearmint as the most commonly used medicinal herbs among California Hispanics [52], and Ortiz and Clauson, who identified chamomile and aloe vera as favored choices among Hispanics residing in Florida [53].

Findings presented in this study show that evidence-based supplement use does not correlate well with specific disease states. Factors such as cultural practices, educational disparities, and healthcare expenses may contribute to this outcome. The selection of specific herbal supplements, which differs from patterns observed in previous research, may reflect the impact of influences such as United States trends or the COVID-19 pandemic.

### Limitations

Limitations for this study included small sample size, language barriers for some participants, cultural expectations in relation to what is considered proper to report, and the utilization of a survey method without in-depth interviews.

## Conclusions

The majority of the supplements used by those with specific diseases were not in alignment with evidence-based guidelines. Therapeutic diets were generally uncommon among participants with these conditions, with the highest prevalence observed in the cardiovascular group. The frequency of herbs and spice use on a weekly basis was similar to that of supplement consumption; however, significant differences in education level and gender were noted among those adhering to special diets. A statistically significant difference (*p* <.001) between male and female participants emerged regarding the use of green tea supplements, as well as all herbs and single vitamins combined. Although special diets were not widely practiced, significant differences were identified between individuals following such diets and those who did not within the disease-specific groups analyzed.

As this is a small sample size, caution is used in the interpretation of the results. Education within this population may be necessary to support evidence-based supplement selection as well as the appropriate use of therapeutic diets. Additional research is warranted to assess supplement knowledge and selection among the Hispanic/Latino/Latina population.
